# Auditory noise increases the allocation of attention to the mouth, and the eyes pay the price: An eye-tracking study

**DOI:** 10.1371/journal.pone.0194491

**Published:** 2018-03-20

**Authors:** Magdalena Ewa Król

**Affiliations:** SWPS University of Social Sciences and Humanities, Faculty in Wrocław, Wrocław, Poland; Harvard Medical School, UNITED STATES

## Abstract

We investigated the effect of auditory noise added to speech on patterns of looking at faces in 40 toddlers. We hypothesised that noise would increase the difficulty of processing speech, making children allocate more attention to the mouth of the speaker to gain visual speech cues from mouth movements. We also hypothesised that this shift would cause a decrease in fixation time to the eyes, potentially decreasing the ability to monitor gaze. We found that adding noise increased the number of fixations to the mouth area, at the price of a decreased number of fixations to the eyes. Thus, to our knowledge, this is the first study demonstrating a mouth-eyes trade-off between attention allocated to social cues coming from the eyes and linguistic cues coming from the mouth. We also found that children with higher word recognition proficiency and higher average pupil response had an increased likelihood of fixating the mouth, compared to the eyes and the rest of the screen, indicating stronger motivation to decode the speech.

## Introduction

Speech is usually presented conjointly with the face of the speaker, while mouth movement is a visual reflection of what is said. In the first six months of life, of all facial regions, infants spend the largest amount of time looking at the eyes of the people around them, reflecting their predominantly social interests [1]. Afterwards, a new pattern eye gaze in response to faces ‘emerges and the mouth area becomes the region of the most intensive scrutiny [2,3]. This change follows the infants’ newfound interest in speech production, as the mouth movements become a source of information useful in disambiguating speech.

This tendency to look at the mouth is reversed in favour of the eyes at some point in childhood [4,5]. It is hypothesized that the change to the adult pattern of preferential fixation on the eyes results from the gain of expertise in their native language', which decreases the requirement for direct access to visual speech information [6]. This is partly reversed again in old age, as older adults fixate more on the mouth region of the face than younger adults [7]. This is caused by compensation for hearing loss or slower speech processing via increasing the reliance on visible speech [8].

Therefore, the increase in mouth fixations under increased speech processing demands should be seen as an adaptive mechanism that provides additional visual cues that are useful in resolving ambiguity in the auditory input. This view is further supported by the research on bilingual children. For example, Pons, Bosch and Lewkowicz [9] reported that the period of preferential looking at the mouth is extended in bilingual infants, helping them to deal with the challenge of acquiring two linguistic codes. Additionally, Lewkowicz and Hansen-Tift [6] demonstrated that attention allocated to the mouth increases when 12-months old infants hear non-native language. These studies indicate that infants attend to the mouth in search of cues to enhance the processing of accompanying speech. Indeed, children with specific language impairment (SLI) were found to allocate more attention to the mouth than typical controls, most likely in order to compensate for their speech processing deficits [10].

Strategic allocation of attention to the mouth under increased speech uncertainty may also be used by adults. For example, Barenholtz, Mavica and Lewkowicz [11] reported that attention to the mouth increased in response to non-native speech in adult participants, but only during a task requiring speech processing. Similarly, adults fixated on the mouth more often when noise was added to the auditory input [12]. This strategy is effective, as the ability to understand speech in difficult conditions is increased when the speaker’s face is visible [13–15].

However, the question is whether this strategy comes at a price. Increasing the share of attention allocated to the mouth decreases the amount that can be allocated to other information. For example, Hosozawa et al. [10] speculated that the strategy to concentrate on the mouth used by children with SLI to compensate for the auditory processing deficit may have a negative impact on their social development. While viewing a nursery song, children with SLI allocated little visual attention to the eyes of the singer, in contrast to the viewing patterns displayed by typically developing (TD) children in the study. So far, however, this hypothesis has found little support, as in the Hosozawa et al. [10] study children with SLI viewed social situations similarly to TD children, and differently from children with Autism Spectrum Conditions (ASC).

Relatedly, a similar hypothesis was proposed with regards to language deficits associated with Autism Spectrum Conditions (ASC). However, the hypothesis that those problems partially stem from the social deficits has not been sufficiently supported. Initially, preferential mouth fixations characteristic for the ASC population were thought to reflect excessive focus on physical aspects of the stimulus, and as such detrimental to socio-communicative development [16–19]. However, Klin et al. [20] reported a positive correlation between social adjustment and preferential looking at the mouth in adults with ASC.

To summarize, the relationship between looking at the mouth and improvements in auditory processing is well established. For this reason, changes in fixations to the mouth are often related to age-related language proficiency, language familiarity, hearing ability and auditory conditions. At the same time, looking at the eyes has been linked to social functioning—increased focus on the eyes correlates with better extraction of social cues and social functioning in general. However, the relationship between social and linguistic skills in typically developing children, mediated by patterns of face processing, is far from clear. Specifically, is there a trade-off between two source of important information- linguistic cues coming from the mouth and social cues coming from the eyes?

In an attempt to better understand that relationship, we studied changes in visual attention to faces of typically developing toddlers in response to increased noise in the auditory input. We assessed the linguistic proficiency of the participants by means of their behavior, using a preferential looking paradigm of Fernald et al. [21], and a standardized inventory of communicative development. We also measured pupil dilation in the task as a reflection of mental effort [22–24], to make sure that increased auditory noise was indeed related to increased processing difficulty, reflected in increased mental effort.

The addition of noise to the auditory stimuli allowed us to use the same speech samples in all conditions, eliminating potential confounds. For example, in the studies comparing patterns of visual attention to faces in response to native and non-native languages, the experimental stimuli differ not only in terms of relative processing difficulty but also novelty [6,11]. To our knowledge, this is the first study investigating changes in the patterns of visual attention to faces induced by the addition of noise to the speech stimuli in toddlers, even though similar studies have been performed in the adult population [12,13].

Our main hypothesis was that adding noise to speech input will lead to shifting fixations from the eyes to the mouth of the speaker. This would represent a trade-off between increasing the ability to understand speech and processing social cues present in the region of the eyes. Obtaining these additional cues from the mouth to resolve auditory uncertainty may come at the price of fixating on the eyes less and forfeiting the social cues that come with it. Thus, we wanted to investigate if the introduction of noise to the auditory input would lead to an increase in the compensatory mouth fixations, and whether that compensation strategy would come at the price of decreased likelihood of fixations to the eyes.

The toddler age range was chosen to show the effect of auditory noise in a group with emerging linguistic skills, and to provide a model of how auditory processing difficulties could impinge on social development via changes in the patterns of visual attention to faces.

## Method

### Participants

A total of 49 toddlers were recruited for the study, but five of this sample were unable to complete the experimental procedure due to general fussiness or unwillingness to take part and/or problems with calibration. The remaining 44 completed the study. Written consent to take part in the study was obtained from the caregivers. In three cases, the experimental data were missing due to a technical malfunction and these cases were completely excluded from all analyses. One additional dataset was excluded from the analysis on the basis of very poor quality data, leaving 40 datasets for the purpose of statistical analysis. Of the remaining 40 children, 21 were girls and 19 were boys. Their mean age was 2.06 years (SD = 0.44) and the age range was from 17 to 35 months. None of the children wore glasses and none had any diagnoses of developmental delays or disorders. None of the mothers reported worrying about any aspect of their child’s development. The study was approved by the Faculty of Psychology II Research Ethics Committee, in accordance with the Declaration of Helsinki.

### Stimuli and design

#### Nursery rhymes task

In the nursery rhymes task, participants were presented with three short video clips of a young woman reciting a short nursery rhyme. Each video clip had a certain level of noise added to the audio track: no noise, medium level of noise or high level of noise. A similar approach, though executed differently, was used in the Vatikiotis-Bateson study on the adult population [12]. In order to introduce the required level of noise, a predetermined proportion of sound samples was randomly selected and substituted with linear interpolation of the neighboring samples. This method allowed us to introduce some noise without garbling the speech, that is preserving the characteristic features of speech. For the medium level of noise the proportion of substituted samples was 5% and for the high level of noise, it was 15%. We asked six adults to rate recordings with different levels of noise on a scale from 1 –very easy to understand to 7 –impossible to understand. Medium level of noise used in our study corresponds roughly to the rating of 3 (quite easy to understand), and high level of noise corresponds roughly to the rating of 5 (difficult to understand). Given that the ratings were provided by adult responders, the chosen noise levels difficulty was likely higher for the toddler population.

The nursery rhymes were composed specifically for the purpose of the study. All consisted of four lines with 8 syllables and an AA BB rhyme scheme. Each nursery rhyme was recited by three young females and recorded on video. The recordings were made on a white background and with a similar level of luminance for all recordings, focusing on the head of the person reciting the poem. Overall, nine video clips were recorded (each person recited all three poems) and each clip was then used to produce video clips with varying levels of noise as described above. This resulted in 27 versions of experimental stimuli of similar length. Each clip lasted approximately 10 seconds.

All stimuli in this task were displayed on a black background. The frame size of each clip was 14.7° x 14.7°, with an average size of the speaker’s face approximately 9° x 12°.

For each person reciting the poems, we created a single-color stimulus with the size identical to the video clips. Its color was obtained by averaging hue and brightness of a sample frame of each video clip. The purpose of this stimulus was to obtain the baseline size of the pupil [25].

#### Looking-while- listening task

This task was based on the looking-while-listening procedure, described by Fernald, Zangl and Portillo [21]. It consisted of thirty trials where two known objects were displayed simultaneously on the screen, while a voice asked the child to look at one of the objects.

The target words in the study were chosen to represent varying levels of difficulty, to avoid a ceiling effect, where older children would know all of the presented words. Ten of the words were taken from the Polish version of the Macarthur CDI: Word and Gestures, ten words were taken from the Words and Sentences, and the final ten slightly more difficult words were added by the experimenters (chosen based on a pilot study). For the list of words and other details of their selection process, see S1 Text.

For each target word “X”, we recorded a sentence: “Where is X”. The sentences were selected for the similarity of intonation, the length of each part of the sentence and the onset of the target word, and finally trimmed to cut out the silent parts using the Praat program [26]. Each sentence lasted 1.5 s. To prevent participants getting bored from exposure to sentences of similar length and intonation, we also recorded ten filler sentences, as suggested by Fernald et al. [21], such as: “Look”, “Can you find it?”, “Will you find it for me?”.

Visual stimuli were 30 full-color photographs representing referents of the target words. The photographs were trimmed to equal size and their levels of brightness and contrasts were equalized. Picture sizes were as close to 9.8° x 9.8° as possible, given the individual shapes of the depicted objects.

#### IRMiK—Polish version of the MacArthur Communicative Development Inventory

Parents of the participants were requested to fill in the IRMiK questionnaire [27], that is the Polish adaptation of the MacArthur Communicative Development Inventory [28]. We used the “Words and Sentences” version, designed for children between 18–36 months. IRMiK is a parent questionnaire assessing different aspects of communicative development, including the assessment of expressive vocabulary.

### Procedure

Parents of the recruited children were requested to fill the IRMiK questionnaire at home, prior to arriving at the laboratory. Upon their arrival to the laboratory, children were seated on their caregiver’s lap. The caregivers were requested to wear a sleeping mask, to prevent them from seeing the computer screen. Children’s eye movements were recorded using a remote eye-tracking device SMI RED250Mobile, at a sampling rate of 60 Hz. The laboratory has no natural light and illumination of the room was kept constant between participants to decrease pupil data variability due to pupillary light reflex.

Participants were seated approximately 70 cm from the computer screen. The experiment was programmed in C# and displayed on a 15” Dell Precision M4800 workstation. Children took part in an in-house 5-point calibration, 4- point validation procedure, where customary dots were replaced with small animated images, accompanied by matching sounds (for example an animated cat and a meowing sound).

To make the experimental procedure less monotonous, both tasks were divided into blocks and interspersed with one another. Every ten trials from the looking-while-listening task were followed by one nursery rhyme.

Each nursery rhyme was preceded by the pupil baseline stimulus displayed for 3 seconds. Each participant watched each of the three nursery rhymes once, each displayed with a different level of noise and read by a different person. The particular version of the clip was randomly selected for each participant and the order of presentation was randomized between participants.

Each block of the looking-while-listening task consisted of ten trials interspersed with attractive filler trials that prevented boredom and loss of attention. Filler trials consisted of 2–3 s. cartoons and animated clips, accompanied by verbal praise. Fillers were displayed after every three experimental trials. Additionally, filler trials were used to check the position of the participants.

Each experimental trial in this task began with a blank screen displayed for 2 s. Next, two pictures (target and distracter) were displayed simultaneously- one to the left and one to the right side of the screen for 2 s. without any accompanying sound. After that, with the pictures still visible, the speech stimulus was presented for 1.5 s., i.e. the question: “where is x?”, where “x” was the name of the target object. Each object was displayed once as the target and once as the distracter. The distracter in each trial was selected at random for each child and the order of target presentation was randomized between participants. After the offset of the question, one of the filler sentences was presented (for example “Can you find it?”), each lasting 1.5 s., followed by 1 s. of silence. Overall, each trial in the task lasted 6 seconds.

## Results

### Data analysis

Fixations and saccades were identified using the SMI Event Detector software, with the default minimum fixation length of 100 ms within a fixation radius of 100 px.

#### The word recognition proficiency

In the looking-while listening task, we defined two Areas of Interest (AOI)–target AOI and distracter AOI. Each was specified as covering the area equal to half of the screen divided vertically, i.e. 640 x 720 pixels (left side and right side, depending where the stimulus was displayed in a particular session). This amounted to 13.8° x 15.9° visual angle. Proportion of total looking time was computed for each AOI for each participant by dividing the amount of time they looked at each AOI, respectively, by the total amount of time they looked at the screen within the trial.

We calculated the proportion of looking time to the target side of the screen for two time periods: before and after the speech onset. After speech onset (M = 0.6, SD = 0.08), the proportion of looking time to the target side of the screen was significantly higher compared to the time period before speech onset (M = 0.5, SD = 0.06), t(34) = -6.32, p<.001, d = 1.07, 95% CI [-0.13, -0.07]. This confirmed that the task was suitable for measuring child’s word recognition ability. In all subsequent analyses, we used only the proportion of looking time to the target side of the screen after the onset of speech, henceforth called the word recognition proficiency score.

To confirm that this measure reflected the level of the child’s linguistic abilities, we correlated it with the child’s vocabulary size reported by the parent. To do that, we calculated the expressive vocabulary score as the total number of words within the IRMiK Words and Sentences questionnaire spoken by the child, as reported by the parent. We found a positive correlation between the expressive vocabulary score and both child’s age (r_s_(35) = .80, p <.001) and the word recognition proficiency score (r_s_(35) = .65, p <.001). We also found a significant correlation between the child’s age and word recognition proficiency score (r = .66, p <.001).

#### The location of fixations

**Fig 1:**
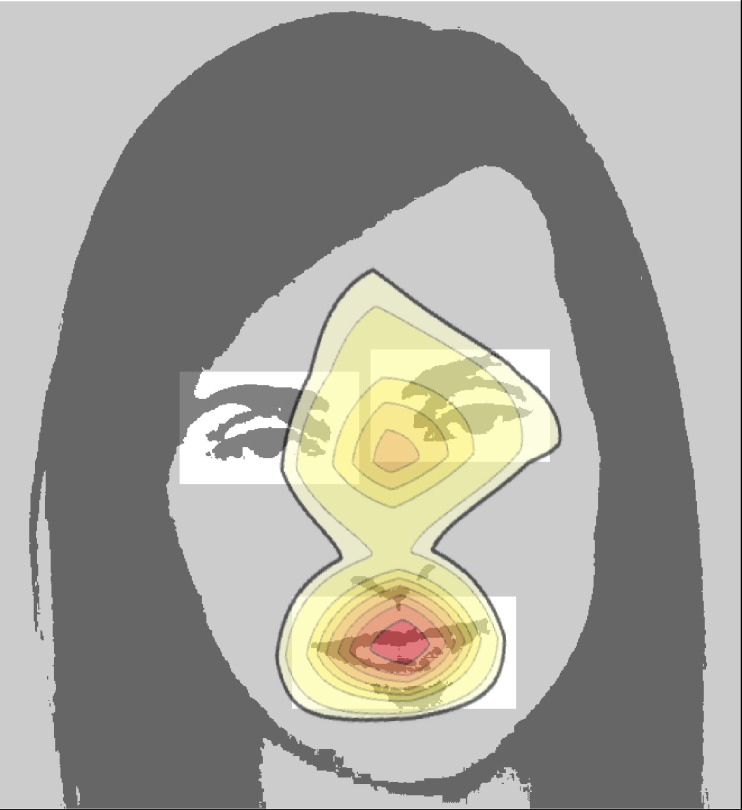
Differential heatmap for patterns of fixations in the high level of noise and no noise conditions. We estimated a smooth kernel distribution with standard Gaussian bandwidth separately for the empirical distribution of fixations in the no noise and high level of noise conditions. Next, we calculated the difference between corresponding probability density functions of the two estimates. The bounded area shows regions where the number of fixations was higher in the high level of noise condition than in the no noise condition. Warmer colors indicate bigger difference. Rectangles represent the position of dynamic eyes and mouth AOIs used in the task. The frame from the clip was filtered to preserve the anonymity of the person in the video.

In the nursery rhymes task, we defined three AOIs- eyes AOI, mouth AOI and the rest of the screen AOI. The eyes AOI was defined as two rectangular areas around the eyes of the speaker, each measuring 3.7° x 2.5° of visual angle. The mouth AOI was defined as a rectangular area around the mouth of the speaker, measuring 5° x 2.5° of visual angle. The position of each AOI was defined on a frame-by-frame basis for every video frame, using the Wolfram Mathematica face detection algorithm. The rest of the screen AOI was defined as all other pixels on the screen. For an example of AOIs position in the nursery rhymes task, see Fig 1. Each fixation within the nursery rhymes task was classified into one of three possible categories, depending on which AOI it was located in: an eye fixation, a mouth fixation and a rest of the screen fixation.

#### The average pupil response

To calculate average pupil response in the nursery rhymes task, we used standard pre-processing procedures [29]. After removing the artifacts (blinks, partial eyelid closures, excessive head movements and loss of tracking) we averaged the pupil size for each fixation. Next, mean pupil sizes were baseline-corrected by subtracting the baseline pupils size obtained from the pupil baseline stimulus, displayed before each nursery rhyme.

### Mixed-effect multinomial logistic regression

To control for the fact that data from multiple observations of the same participant are likely to be correlated, we estimated a mixed-effects multinomial logistic regression [30] using IBM SPSS Statistics 22.0 software. Mixed-effects models reveal patterns common to all participants while controlling for variance caused by individual differences. Another advantage over repeated-measures approaches is that mixed-effects models make it possible to use datasets in which observations are missing at random. This is especially important in studies of young children, because the experiment duration is limited by their attention span and the quality of eye-tracking or EEG data is usually lower than for adult participants, resulting in a significant proportion of missing data. Although this type of modelling is relatively new, it is frequently used in developmental psychology research, and especially in developmental psycholinguistic [31–33]. Finally, a study by DiGiorgio et al. [34] on whether faces capture and retain attention of infants aged 3 and 6 months, and a study by Guillon et al. [35] on atypical attentional orienting towards faces in ASC toddlers, are two good examples of research studies on gaze patterns in children (infants and toddlers respectively) using mixed-effect modelling.

For each fixation, the dependent variable was the fixation target: mouth—eyes—or the rest of the screen (the regression’s reference category was the mouth). The independent variables were the child’s age, word recognition proficiency, average pupil response (as a measure of mental effort related to increased auditory noise) and the noise level (with noise = 0 as the reference category). Thus, the resulting regression models the likelihood of looking at either the eyes or the rest of the screen rather than the mouth, depending on the values of the predictor variables.

Given that the child’s age and word recognition proficiency are strongly correlated (r = .66, p <.001), we performed multicollinearity diagnostics and obtained variance inflation factor (VIF) for age = 1.77 and word recognition proficiency score = 1.79, which in both cases is below the threshold of 2. This means that multicollinearity was unlikely to be a problem, and both variables were included in the regression.

The observations were grouped by both participant and stimulus that was observed (as each video was seen only once by a given participant, this was equivalent to grouping by participant / trial). Thus, we allowed for the fact that fixations carried out by the same subject might be correlated, particularly if they also occurred in the same trial. Furthermore, we allowed for random regression slopes in addition to a random intercept, i.e. for the fact that the relationship between pupil dilation and noise on the one hand and fixation target on the other could vary between subjects depending on their age and language proficiency. In assessing the statistical significance of the fixed (overall) effect of noise on looking patterns, we therefore took into account its variance across participants. The estimates of the resulting two-level multinomial logistic regression are presented in Table 1.

**Table 1:** The fixed effect estimates of the mixed-effects multinomial logistic regression, with the likelihood of looking at the eyes rather than the mouth and at the rest of the screen rather than the mouth modelled as a function of the noise level, word recognition proficiency score, age and average pupil response.

	b	SE		t		p	95% CI
	Eyes vs. mouth						
Fixed effect							
Intercept	1.90	0.67		2.84		.01	[0.59, 3.21]
Age	0.63	0.29		2.22		.03	[0.07, 1.19]
WRPS	-5.19	1.51		-3.43		.001	[-8.15, -2.22]
Random effect							
Noise level = 3	-0.55	0.22		-2.52		.01	[-0.97, -0.12]
Noise level = 2	-0.44	0.22		-1.99		.05	[-0.88, -0.01]
APR	-0.18	0.07		-2.71		.01	[-0.31, -0.05]
	Rest of the screen vs. mouth						
Fixed effect							
Intercept	1.76		0.51		3.42	.001	[0.75, 2.76]
Age	0.43		0.22		2.00	0.05	[0.01, 0.86]
WRPS	-3.04		1.15		-2.65	.01	[-5.28, -0.79]
Random effect							
Noise level = 3	-0.56		0.17		-3.32	.001	[-0.88, -0.23]
Noise level = 2	-0.07		0.17		-0.42	.68	[-0.40, 0.26]
APR	-0.28		0.05		-5.33	<.001	[-0.37, -0.17]

Notes: WRPS = Word Recognition Proficiency Score, APR = Average Pupil Response.

We found no significant effect of age in either of the two comparisons: between the likelihood of looking at the eyes rather than the mouth and looking at the rest of the screen rather than the mouth. However, there was a significant effect of word recognition proficiency in both comparisons, i.e. children with higher word recognition proficiency scores were more likely to look at the mouth of the speaker. There was also a significant effect of average pupil response, i.e. children with a larger pupil response were more likely to look at the mouth. Finally, we found significant effects of both levels of noise on the likelihood of looking at the eyes rather than the mouth. For both levels of noise, compared to the no noise condition, the likelihood of looking at the mouth rather than the eyes was higher. Similarly, for the high level of noise, the likelihood of looking at the mouth rather than the rest of the screen was higher than under no noise. However, the effect of the medium level of noise was not significant in this case.

### Proportion of mouth and eyes fixations depending on the noise level

**Fig 2:**
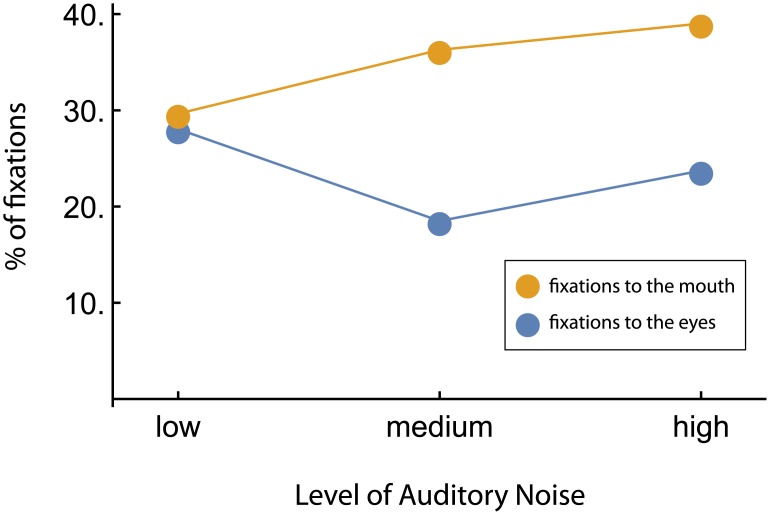
Proportion of fixations to the mouth and to the eyes depending on the noise level.

When performing ANOVA, six cases had to be excluded due to missing data, as ANOVAs cannot handle cases when all data in one condition are missing. A 2 (face part: eyes vs mouth) x 3 (noise level: no noise, medium noise, high noise) repeated-measures ANOVA was performed. I found a significant effect of face part (F(2,68) = 10.95, p = .002, η_p_^2^ = .24, significantly more fixations to the mouth), noise level (F(2,68) = 4.51, p = .01, η_p_^2^ = .12) and a significant face part x noise level interaction (F(2,68) = 6.81, p = .002, η_p_^2^ = .17. Significant interaction was followed by planned repeated contrasts, which revealed a significant difference between no noise and medium noise in fixations to the mouth and to the eyes (F(1,34) = 6.89, p = .01, η_p_^2^ = .17, but no significant differences in the pattern of looking at the mouth and the eyes between medium an high levels of noise (F(1,34) = 0.81, p = .38, η_p_^2^ = .02 (Fig 2).

## Discussion

The purpose of the study was to investigate the changes in the patterns of visual attention to faces in response to auditory noise in speech input. We hypothesized that auditory noise would increase the uncertainty of speech input and in consequence participants will turn to visual sources of speech cues to resolve that ambiguity. This will be reflected in the increase of likelihood of fixations to the mouth area. Additionally, we hypothesized a trade-off between fixations on the eyes and the mouth. As a result of this trade-off, higher levels of auditory noise would lead to a decrease in the likelihood of fixations in the eyes area. Our results confirm these hypotheses, as we found a noise-related increase in the likelihood in the fixations on the mouth area, compared to the eyes area.

The effect of increased mouth fixations induced by an increase in speech-related difficulty or uncertainty is well established. Evidence from studies ranging from the language acquisition in both typically and atypically developing children [6,10,36–38], studies on bilingualism [9,11], or older adults [7] to experiments investigating speech processing in adverse conditions in adults [12,13,39,40] support the view of the adaptive role of mouth fixations in adverse listening conditions. The results of this study also provide further evidence for this view. We found an increase in the likelihood of fixations to the mouth area in the medium and high level of noise conditions, compared to the no noise conditions. This is also evidence for top-down strategic and task-oriented eye-movement control in toddlers, who actively change their looking patterns to accommodate to the task and current conditions [41].

We also hypothesized that there would be a trade-off between the fixation times in the mouth and the eyes areas. In other words, the shift of attentional resources to the mouth may come at the price of decreasing the attentional share allocated to another concurrent task, primarily the monitoring of the eyes in search of social cues. We found that the increased likelihood of fixations to the mouth, comparing both mouth to the eyes and mouth to the rest of the screen. This effect was significant for both medium and high levels of noise in case of the mouth-eyes comparison. In case of the mouth-rest of the screen comparison, it was present only for the high level of noise. This pattern of results indicates that the increased attention to the mouth of the speaker in adverse speech processing conditions comes at the price of both decreased attention to the mouth and decreased attention to other parts of the stimulus. In other words, when the noise increases a little, the child’s visual attention shifts from the eyes to the mouth. When the noise increases a lot, the child’s visual attention shifts from both eyes and the rest of the stimulus to the mouth. Therefore, these results confirm the presence of the trade-off between the eyes and the mouth, that is the sources of social and linguistic cues.

Given the evidence that the eyes provide important social cues [42,43], the trade-off described in this study could have potentially detrimental effects on the development of certain groups of children. For example, this could lead to secondary social deficits resulting from decreased attention to nonverbal social cues present in the eye area of the face in children with auditory processing difficulties, such as children with SLI diagnoses, as hypothesized by Hosozawa [10]. Or, on the other hand, the existence of such an effect could contribute to the explanation of problems with eye-contact in children with ASC, who often are linguistically-impaired. However, given the complexity of the face processing research in the ASC population, the generalizability of these results to the ASC group is very uncertain. For example, Klin et al. [20] found that ASC participants generally had less fixations to the eyes, but those of them who fixated more on the mouth, functioned better socially, while those who fixated more on the objects, had lower social functioning. The reason for the correlation between mouth and social functioning could be that in ASC participants, interest in the mouth reflected interest in what was being said, i.e. a social interest, compared to non-social interest in the objects. Finally, gaze avoidance in the ASC population is not necessarily connected to language processing at all—in fact, there is plenty of evidence it may be linked to hyperarousal of the autonomic response [44,45], mediated by the amygdala [46].

This analysis is purely speculative and we do not know whether such decrease in the eyes fixations could be observed outside of laboratory conditions in children with auditory processing difficulties. Additionally, even if that was the case, we do not know whether this effect is significant enough to lead to any long-term disadvantages in social cues monitoring. Finally, it is important to note that adding noise to the speech input has not been shown to be a proxy for language processing difficulties, characteristic of SLI or ASC. Hayiou-Thomas, Bishop and Plunkett [47] showed that compressing speech to 50% of its original rate produced patterns of errors in typically developing children similar to those characteristic of SLI. However, this is a very different type of manipulation than adding noise to the speech input, so this needs to be taken into consideration when interpreting the results.

Furthermore, we found a significant effect of word recognition proficiency on the likelihood of fixations to the mouth. The results indicate that children with higher scores in the word recognition task were more likely to look at the mouth compared to both the eyes and the rest of the screen. In other words, we find that interest in speech, reflected in the focus on the mouth, is related to better linguistic ability. A similar positive relationship between preferential looking at the mouth and linguistic skills has been previously shown by Norbury et al. [48] and Young et al. [49], albeit in different populations and age groups. This leads to an interesting possibility that intrinsic motivation and interest in different types of stimulation can change the speed of learning of different skills- or to put it simply, we become good at what we enjoy doing. For example, decreased social interests in children with autism may lead to missing opportunities of learning in the social domain [50]. However, the alternative and perhaps more parsimonious explanation is that children with higher levels of language skills were more motivated to understand the speaker because the task was better suited to their skills. For children with a lower level of linguistic skills, the speech in the task could have been less comprehensible and therefore, they were less motivated to focus on the speech. This result may seem contradictory to the main hypothesis, i.e. that increased looking at the mouth is related to linguistic difficulties. Indeed, that increased linguistic proficiency at some point in childhood leads to a decrease in mouth fixations. However, before this point, increased fixations to the mouth are common in toddlerhood (the age we studied), in response to the developmental task at hand- i.e. language acquisition. So, in general, we would expect higher linguistic ability to be related to decreased mouth fixations- but actually, toddlerhood may be an exception to the rule. Increased mouth fixations at this developmental stage may actually be related to higher linguistic ability, as a sign of interest in language. This is what was found in the study- toddlers who fixated more on the mouth had better WRPS, but if an older, linguistically proficient group was tested, this relationship could be reversed. This can be observed in the data as older children looked at the mouth less than younger children, after excluding the effect of language ability, so the shift from the mouth looking to the eyes looking may have already started in the oldest participants in this group.

Finally, we found a significant effect of the average pupil response on the likelihood of fixation to the mouth. Children with larger average pupil response were more likely to fixate the mouth, compared to both the eyes and the rest of the screen. Larger pupil response implies higher mental effort [22,24], so this result suggests that children, who looked at the mouth more, were putting more effort in processing the speech. Children, who looked at the mouth less often, may have been less interested in processing and understanding the speech.

To summarize, higher levels of auditory noise-induced an increase in the likelihood of mouth fixations, at the price of fixating both the eyes and the rest of the screen less often. When the level of noise was moderate, only the eyes-mouth trade-off was significant, while for the high level of noise, the shift of attention to the mouth was related to both decrease in the eyes and the rest of the screen fixations.

Therefore, this study provides evidence for the trade-off between attending to social and linguistic cues. This suggests that shifting attention to the mouth under adverse listening conditions is an adaptive strategy, which requires a sacrifice in concurrent gaze monitoring. Whether this trade-off is present outside of laboratory conditions in children with auditory processing difficulties remains an open question.

Additionally, this is the first study using stimuli, that differ only in one respect (noise level) and that do not differ in terms of their linguistic or other properties (for example, familiar vs unfamiliar language) to test for adaptive increase in attention to the mouth in children. The only other study of this kind was performed by Vatikiotis-Bateson et al. [13], but on the adult population. Finally, this is the first study showing the mouth-eyes trade-off, first hypothesised by Hosozawa et al., who postulated its existence in children with SLI. The study can thus be perceived as a “proof of existence” of the eyes—mouth trade-off in face-processing, mediated by auditory difficulty.

## Supporting information

S1 Text List of words used in the looking-while-listening task. (DOCX)

S1 Dataset Fixation locations, noise level, participants’ language scores, pupil dilations, ages. (CSV)
