# Supplementary material for: Auditory noise increases the allocation of attention to the mouth, and the eyes pay the price: An eye-tracking study
Source: PLoS One. 2018 Mar 20;13(3):e0194491. doi: 10.1371/journal.pone.0194491 (PMC5860771; doi:10.1371/journal.pone.0194491)
Supplement: S1 Text — (DOCX) [file pone.0194491.s001.docx]

# Supporting Information

### List of words used in the looking-while-listening task.

| **No** | **Word** | **Translation to English** | **Source** |
| --- | --- | --- | --- |
| 1 | Banan | Banana | Words and Gestures |
| 2 | Jabłko | Apple | Words and Gestures |
| 3 | Balon | Balloon | Words and Gestures |
| 4 | Piłka | Ball | Words and Gestures |
| 5 | Szalik | Scarf | Words and Gestures |
| 6 | Czapka | Hat | Words and Gestures |
| 7 | Drzewo | Tree | Words and Gestures |
| 8 | Kwiatek | Flower | Words and Gestures |
| 9 | Lampa | Lamp | Words and Gestures |
| 10 | Krzesło | Chair | Words and Gestures |
| 11 | Groszek | Pea | Words and Sentences |
| 12 | Keczup | Ketchup | Words and Sentences |
| 13 | Prezent | Present | Words and Sentences |
| 14 | Gumka | Eraser/ rubber | Words and Sentences |
| 15 | Bluzka | Blouse | Words and Sentences |
| 16 | Pasek | Belt | Words and Sentences |
| 17 | Gwiazda | Star | Words and Sentences |
| 18 | Chmura | Cloud | Words and Sentences |
| 19 | Czajnik | Kettle | Words and Sentences |
| 20 | Młotek | Hammer | Words and Sentences |
| 21 | Czosnek | Garlic | Selected by the author |
| 22 | Kiwi | Kiwi | Selected by the author |
| 23 | Globus | Globe | Selected by the author |
| 24 | Bączek | Spinning Top | Selected by the author |
| 25 | Broszka | Brooch | Selected by the author |
| 26 | Krawat | Tie | Selected by the author |
| 27 | Hydrant | Hydrant | Selected by the author |
| 28 | Róża | Rose | Selected by the author |
| 29 | Wieszak | Hanger | Selected by the author |
| 30 | Śpiwór | Sleeping Bag | Selected by the author |
